# Effects of a Single Session of OnabotulinumtoxinA Therapy on Sleep Quality and Psychological Measures: Preliminary Findings in a Population of Chronic Migraineurs

**DOI:** 10.3390/toxins15090527

**Published:** 2023-08-27

**Authors:** Angelo Torrente, Paolo Alonge, Laura Pilati, Andrea Gagliardo, Lavinia Vassallo, Vincenzo Di Stefano, Antonino Lupica, Irene Quartana, Giovanna Viticchi, Mauro Silvestrini, Marco Bartolini, Cecilia Camarda, Filippo Brighina

**Affiliations:** 1 Department of Biomedicine, Neurosciences and Advanced Diagnostics, University of Palermo, 90127 Palermo, Italy; angelo.torrente@unipa.it (A.T.); alongep95@gmail.com (P.A.); laura.pilati.91@gmail.com (L.P.); andrigl@gmail.com (A.G.); lavinia.v1994@gmail.com (L.V.); vincenzo19689@gmail.com (V.D.S.); antlupica@gmail.com (A.L.); irenequartana@gmail.com (I.Q.); cecilia.camarda@unipa.it (C.C.); 2 Clinical Neurophysiology Unit, Sleep Lab, “Clinical Course”, 90143 Palermo, Italy; 3 Neurological Clinic, Marche Polytechnic University, 60020 Ancona, Italy; viticchi.g@gmail.com (G.V.); m.silvestrini@univpm.it (M.S.); m.bartolini@univpm.it (M.B.)

**Keywords:** onabotulinumtoxinA, chronic migraine, sleep, sleep quality, migraine prevention, patient-related outcome measures

## Abstract

Chronic migraine is a burdensome condition, and onabotulinumtoxinA is revealed to be an effective therapy. Migraine shows a bidirectional relationship with sleep, but the effects of preventive therapies on sleep quality are poorly studied. This study aims to evaluate the effects of a single session of onabotulinumtoxinA on patients’ sleep quality and correlates the results with measures of comorbid anxiety/depression. Patients completed self-administrable questionnaires about sleep quality (Pittsburgh Sleep Quality Index—PSQI) and psychological symptoms (Beck Depression Inventory, 2nd edition—BDI-II—and Hospital Anxiety and Depression Scale—HADS—subscales “a” and “d” for anxiety and depression, respectively), and reported migraine frequency at baseline and after 12 weeks. The 42 included patients showed a significant reduction in migraine days (from 20.6 ± 6.0 to 13.6 ± 6.2, *p* < 0.001), while no changes were observed in sleep quality (PSQI score from 11.0 ± 5.0 to 9.8 ± 4.6, *p* = 0.277) or psychological measures (BDI-II from 16.7 ± 10.2 to 15.7 ± 10.3, *p* = 0.678; HADS-a from 10.3 ± 4.8 to 9.3 ± 5.5, *p* = 0.492; and HADS-d from 7.2 ± 3.9 to 7.1 ± 5.0, *p* = 0.901). On the other hand, a strong correlation among PSQI, BDI-II, HADS-a, and HADS-d scores (*p* < 0.001, rho > 0.7) was found. Despite its efficacy in migraine prevention, a single session of onabotulinumtoxinA was not able to affect patients’ sleep quality or their psychological symptoms.

## 1. Introduction

Migraine is a very burdensome disorder, characterized by repeated episodes of moderate-to-severe headache associated with unpleasant sensations such as nausea and/or light and noise intolerance, limiting daily living. Moreover, it is, to date, general knowledge that migraineurs’ brains show permanent modifications even among attacks (i.e., interictally) due to constant cortical hyperexcitability or alterations in sensory processing, as demonstrated by several neurophysiological studies [1,2]. On a clinical level, several migraineurs often report interictal cognitive (i.e., attention or memory impairment) and/or psychological (i.e., anxiety or depressive) symptoms [3,4,5]. Headache frequency is variable in migraineurs, but sometimes it is possible to recognize factors that may worsen or trigger pain, such as stress, weather changes, alcohol or, in women, the premenstrual period [6]. Another identified factor influencing migraine is represented by sleep, as sleep disturbances usually worsen migraine [6,7]. The relation between sleep and migraine is deeper and more complex than it might seem, since both sleep deprivation and excessive sleep may trigger headache, but, conversely, several patients find relief from sleeping during an attack [7,8]. On the other hand, the persistence of headache overnight or the nocturnal onset of migraine attacks can lead to poor sleep quality in migraineurs, configuring a bidirectional relationship [9]. Moreover, different sleep habits (i.e., chronotype) seem to influence migraine frequency, too [10]. Despite the fact that it is well-known that anxiety and depression are among the most recognized detrimental factors of sleep quality [11], some studies highlighted how migraineurs’ poor sleep quality could not be entirely explained just by comorbid anxiety or depression compared to nonmigraineur subjects [12].

When headache frequency increases (i.e., chronification), reaching or exceeding a threshold of 15 days per month (among which at least 8 days present migraine characteristics or lead to an acute medication intake) for more than three months, it is possible to formulate the diagnosis of chronic migraine (CM) [13]. CM is by far a worse condition than episodic migraine (EM—i.e., when the above-mentioned frequency criteria are not satisfied), which is associated with detrimental mechanisms (e.g., central and peripheral sensitization), making it harder to manage [14,15]. As a matter of fact, CM is associated with more pronounced neurophysiological changes [1,16], as well as worse psychological symptoms and disability than EM [17,18]. One of the first approved therapies for CM based on its pathophysiological changes was onabotulinumtoxinA (or botulinum neurotoxin A—BoNT-A), since it is believed to counteract peripheral and central sensitization mechanisms underlying chronification [19]. Following the Phase 3 REsearch Evaluating Migraine Prophylaxis Therapy (PREEMPT) protocol (in muscle injections of 155–195 international units (IU) divided among 31–39 cranio-cervical sites every 12 weeks) patients can find relief in both the reduction of migraine frequency and the impact on their lives even after the first therapy session [20,21]. Moreover, a positive effect on psychological symptoms associated with CM has been demonstrated [22], but the effects of this treatment on sleep quality have been poorly studied to date. The primary objective of the present study is to evaluate any changes on perceived sleep quality after one session of BoNT-A using the PREEMPT follow-the-pain protocol in patients affected by CM. In addition, the effects on headache frequency and its associated psychological symptoms are also investigated.

## 2. Results

### 2.1. Population

In the period of observation, 42 (32 females and 10 males) patients were accepted to be included in this study. Following the inclusion and exclusion criteria, no subject presented any previous sleep disorder diagnosis or any neurological condition other than CM. Among the included patients, two (4.8%) reported a clinical presentation reflecting the diagnosis of migraine with aura. Demographic characteristics and the main clinical features regarding migraine are reported in Table 1. Most patients used oral acute medications, such as triptans (64.3%) or nonsteroidal anti-inflammatory drugs (NSAIDs—35.7%). The complete list of the acute drugs used may be found in Table 1.

### 2.2. Headache Features

**Figure 1:**
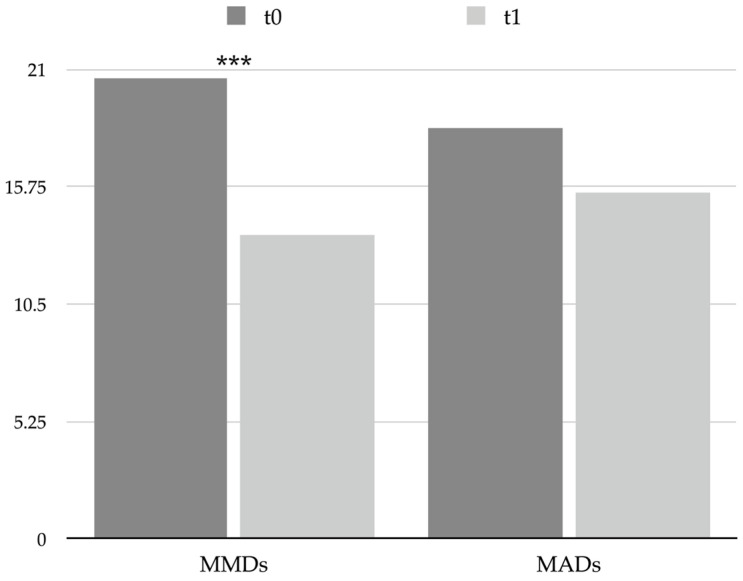
Graphical representation of headache frequency and acute drug use at t0 and t1. *** = *p* < 0.001. Abbreviations: MADs: monthly acute medication days; MMDs: monthly migraine days.

Patients at t0 presented a high number of monthly migraine days (MMDs), which decreased significantly after a single session of BoNT-A (from 20.6 ± 6.0 at t0 to 13.6 ± 6.2 at t1, *p* < 0.001; see Table 2 and Figure 1), with 27 patients (64.3%) identified as responders and 15 (35.7%) as nonresponders, thus reflecting the efficacy of the treatment in preventing migraine attacks.

Nevertheless, the number of monthly acute medication days (MADs) diminished slightly (from 18.4 ± 7.7 at t0 to 15.5 ± 10.1 at t1) without reaching statistical significance (see Table 2 and Figure 1). Furthermore, several patients met the International Classification of Headache Disorders 3rd edition (ICHD-3) criteria for medication overuse headache (MOH) at t0 (35, 83.3%), and according to the reduction in MADs, even the number of MOH patients decreased at t1 (27, 64.3%, chi-square < 0.001).

Lastly, the BoNT-A injective therapy was well tolerated, and the only reported side effects were constituted by a migraine attack slightly more severe than usual lasting 2 days after the procedure in three patients (7.1%) and mild cervicalgia during the afternoon after the procedure in two patients (4.8%).

### 2.3. Sleep Quality

Overall, patients’ sleep quality was not significantly influenced by the single session of therapy with BoNT-A, as no significant change in the Pittsburgh Sleep Quality Index (PSQI) score was observed (from 11.0 ± 5.0 at t0 to 9.8 ± 4.6 at t1; see Table 3). Nevertheless, the number of patients reporting poor sleep quality at baseline (39, 92.9%) was significantly reduced (35, 83.3%) at t1 (chi-square = 0.017).

### 2.4. Psychological Correlates

**Figure 2:**
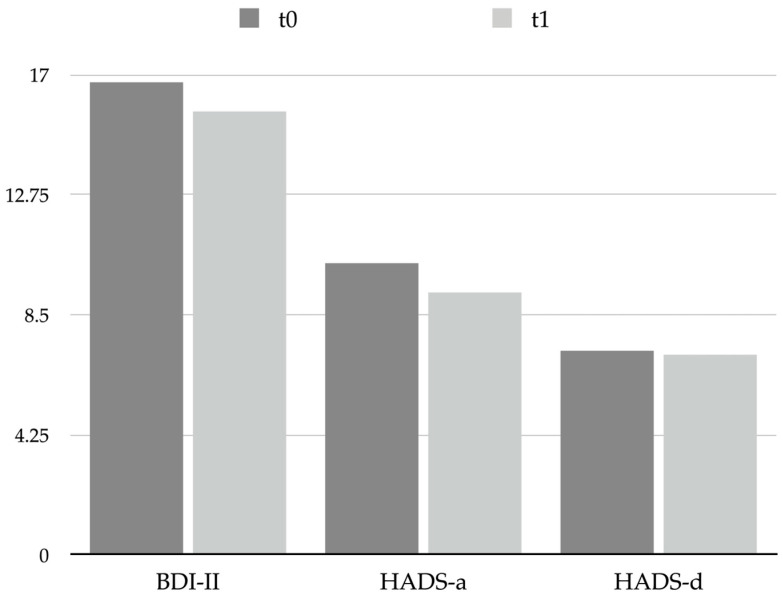
Graphical representation of psychological scales scores at t0 and t1. Abbreviations: BDI-II: Beck Depression Inventory, 2nd edition; HADS: Hospital Anxiety and Depression Scale; HADS-a: anxiety subscale; HADS-d: depression subscale.

No significant changes were observed between t0 and t1 regarding the scores of the questionnaires used to evaluate depression and anxiety symptoms, which were the Beck Depression Inventory, 2nd edition (BDI-II) and Hospital Anxiety and Depression Scale (HADS), subscales for anxiety (HADS-a) and for depression (HADS-d): BDI-II score changed from 16.7 ± 10.2 at t0 to 15.7 ± 10.3 at t1, *p* = 0.678; HADS-a score changed from 10.3 ± 4.8 at t0 to 9.3 ± 5.5 at t1, *p* = 0.492; and HADS-d score changed from 7.2 ± 3.9 at t0 to 7.1 ± 5.0 at t1, *p* = 0.901 (see Table 4 and Figure 2); thus, a single session of BoNT-A was revealed to be not effective in influencing patients’ psychological affections. It is worth noticing that the mean scores obtained reflect a mild level of psychological symptoms in CM patients (i.e., depression measured by BDI-II and anxiety measured by HADS-a) both at t0 and at t1.

### 2.5. Impact and Disability

**Figure 3:**
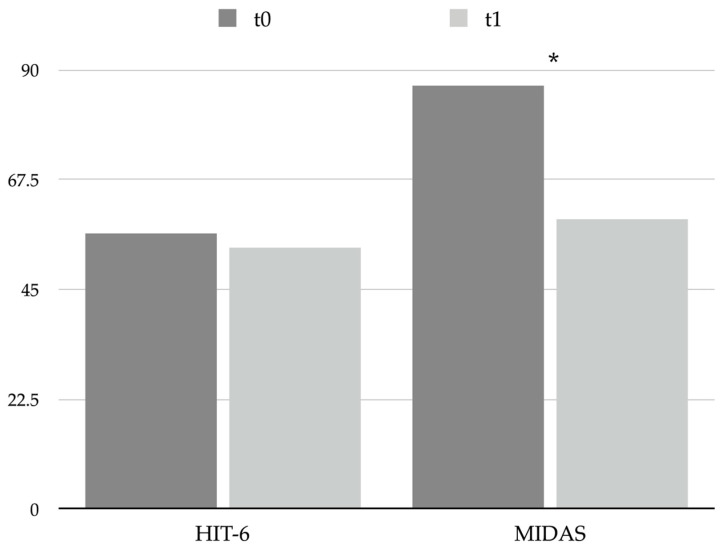
Graphical representation of headache impact and disability questionnaires scores at t0 and t1. * = *p* < 0.05. Abbreviations: HIT-6: Headache Impact Test 6; MIDAS: Migraine Disability Assessment.

BoNT-A was revealed to be effective in reducing migraine-associated disability significantly (Migraine Disability Assessment—MIDAS—score changed from 86.8 ± 64.4 at t0 to 59.3 ± 42.9 at t1, *p* = 0.033; see Table 5 and Figure 3), even if patients’ mean MIDAS score pointed out a severe grade of disability at both t0 and t1. On the other hand, no substantial changes in the perceived impact of headache on everyday life were observed (Headache Impact Test 6—HIT-6—scores changed from 56.4 ± 12.5 to 53.7 ± 17.5, *p* = 0.520; see Table 5 and Figure 3).

### 2.6. Correlation Analysis

The Spearman correlation analysis showed significant relations among several of the studied parameters; in particular, a highly significant correlation was found (*p* < 0.001) between HADS-a and BDI (rho = 0.866), HADS-a and HADS-d (rho = 0.810), HADS-d and BDI (rho = 0.804), PSQI and BDI-II (rho = 0.772), PSQI and HADS-d (rho = 0.736), PSQI and HADS-a (rho = 0.723), PSQI and MIDAS (rho = 0.540), MMDs and MIDAS (rho = 0.507), PSQI and MMDs (rho = 0.443), MMDs and MADs (rho = 0.422), and MIDAS and HIT-6 (rho = 0.416); the remaining significant correlations may be found in the Supplementary Material (see Supplementary Figure S1). The mentioned results indicate a strong direct correlation among sleep quality, anxiety, and depression, as a clinical worsening of the latter ones is associated with a deterioration of the former and vice versa. Furthermore, a weaker correlation was found between sleep quality and migraine associated disability as well as migraine frequency. Lastly, as expected, psychological measures scores strongly correlated among themselves.

## 3. Discussion

Good sleep quality is a fundamental requirement for a healthy life, since it is associated with good mental health [11,23]. Several factors may affect sleep quality nowadays, with anxiety and depression being among the most frequent ones [24,25]. Recent events revealed how even the SARS-CoV-2 pandemic, with its associated social distancing regulations, affected sleep negatively [26,27]. Sleep, in many ways, is one of the main factors influencing primary headaches, as demonstrated from studies performed on cluster headache [28]. On the other hand, people who refer poor sleep quality show an increased frequency of tension-type headache and an increased impact of headache on everyday life [29,30]. Regarding migraine, the relation seems to be bidirectional, since a poor sleep quality may lead to an increase in headache frequency [31] as well as a high headache frequency (with pain occurring or perduring overnight) may worsen sleep quality [32]. Moreover, sleep quality is negatively affected by migraine chronification, as sleep alterations are more evident in CM patients [9,33,34]. Indeed, a previous study by our group demonstrated that more than 60% of CM patients reported poor sleep quality (i.e., a score >5 on the PSQI questionnaire) [35]. Since migraine frequency and sleep quality show an indirect correlation (i.e., when the frequency of pain episodes increases, sleep quality worsens), one could speculate that an effective migraine prevention may lead to a concomitant sleep improvement. Actually, the same above-mentioned research pointed out how, despite a progressive significant reduction in MMDs, sleep quality measured by PSQI did not show any significant variation in CM patients treated with erenumab, an anticalcitonin gene-related peptide monoclonal antibody [35]. A recent work of this group demonstrated how a single session of BoNT-A was able to reduce migraine frequency and even partially restore some of neurophysiological alterations to multisensory integration shown by CM patients [36]. Even the present study confirmed that after a single therapy session, more than 60% of patients found relief in migraine frequency (i.e., responders), reducing even acute medication intake (even if not significantly) and the number of patients showing MOH features. Despite the general improvement in headache features and the moderate correlation found between MMDs and PSQI scores, no significant change in overall sleep quality was observed as measured with the PSQI questionnaire; the only improvement that is worth mentioning is about the reduction in the percentage of patients who were classified as reporting poor sleep quality (from 92.9% at baseline to 83.3% at t1). It is possible that any definite influence of the preventive therapy on sleep may be detectable after a more extended period of treatment; thus, the present preliminary data from a single session of therapy are revealed to be not sufficient. As a matter of fact, results from the Chronic Migraine OnabotulinuMtoxinA Prolonged Efficacy open-Label (COMPEL) study demonstrated how a significant reduction in PSQI score, revealing an improvement in sleep quality, was appreciated after 108 weeks of treatment [37]. Another study that investigated the effects of BoNT-A therapy on sleep quality demonstrated that after 48 weeks of follow-up, overall sleep quality of the whole population did not change, but sleep quality improved in patients who did not show a negative emotional state at baseline [38]. Indeed, the emotional state is one of the main factors influencing sleep, as demonstrated even by the correlation analysis performed in the present study: PSQI scores showed a very strong positive correlation with BDI-II, HADS-d, and HADS-a scores, suggesting how sleep quality strongly depends on patients’ anxious and depressive symptoms.

Data in the literature seem to suggest that after 3 months from the first BoNT-A session, CM patients show a positive effect on depressive symptoms but not on anxiety ones [39]. Nonetheless, the patients from the present cohort reported no significant changes in the psychological measures studied. It is plausible that an improvement in migraine-correlated psychological symptoms could be observable after more prolonged use of the preventive therapy, as demonstrated by long-term studies on BoNT-A [37]. CM is a disorder that affects patients’ lives in many ways, causing severe disability in daily activities and in work ones as well as influencing the psychological sphere [40]. It is understandable that such a precipitate, vicious circle can establish itself and persist even over several years, and, despite specific and effective treatments (e.g., BoNT-A), the global return to a pre-CM condition is a long and hard journey that must be undertaken by the patient together with the clinician. Having said that, it is understandable that some aspects may change more rapidly, such as headache frequency and related disability (see significant change in MIDAS score of the present study), while others need a longer interval (e.g., psychological and sleep measures scores). Furthermore, since the presented preliminary data show a stronger correlation between sleep quality and psychological measures than between sleep quality and MMDs, it is possible that therapy continuation and future follow-ups may reveal an improvement in psychological correlates and concurrent sleep quality.

The main limitations of the present study are represented by the short period of observation and by the use of a single patient-reported outcome measure to study sleep quality. Future research directions should be oriented towards widening the studied population and the follow-up period. Even if the included patients denied any previous sleep disorder diagnosis, neither a sleep disorders battery test nor an extended sleep evaluation was performed at baseline. Furthermore, the study of single aspects of sleep quality (e.g., sleep efficiency) or of sleep-related daytime symptoms may be of great value to better understand the mutual influences between migraine and sleep. Lastly, the use of specific techniques such as actigraphy or polysomnography could provide reliable tools to obtain stronger results.

## 4. Materials and Methods

### 4.1. Study Procedures

The present study protocol was conducted following the principles contained in the Declaration of Helsinki [41]. Before enrolment, we asked for and obtained approval from the Palermo I Ethics Committee, University of Palermo, Palermo, Italy. Informed consent was acquired from patients before they were included.

### 4.2. Participants

Patients referred to the headache outpatient clinics of the “Paolo Giaccone” University Hospital, Palermo, Italy, and of the Azienda Ospedaliero-Universitaria delle Marche, Ancona, Italy, were enrolled in the presence of the following inclusion criteria: (i) age >18 years; (ii) diagnosis of CM according to ICHD-3 criteria [13]; (iii) indication to start preventive treatment with BoNT-A [42]; (iv) stable dosage of other medications taken, including other eventual preventive treatments for migraine within the previous 3 months; and (v) signing of informed consent. The exclusion criteria were constituted by (i) a clear diagnosis of sleep disorders (e.g., insomnia or obstructive sleep apnea syndrome); (ii) a diagnosis of major depression or any other psychiatric comorbidity; and (iii) chronic intake of any drug known to affect sleep (e.g., benzodiazepines, amitriptyline, or melatonin) or prescribed for a sleep disorder.

### 4.3. Study Design

This is a prospective observational study evaluating the impact of a single session of BoNT-A preventive treatment on sleep quality and other psychological correlates in chronic migraineurs. After signing of the informed consent, patients were evaluated with self-administered questionnaires on the day of the first treatment session (t0) and after 12 weeks (t1). Patients (who were already taken in care at the headache outpatient clinics) reported in a specific, previously provided headache diary regarding the 3 months before the therapy start and kept on filling it out during the whole investigation.

### 4.4. Preventive Treatment Protocol

The preventive treatment with BoNT-A was provided according to the PREEMPT follow-the-pain protocol, administering 195 IU of drug every 12 weeks [20]. The procedure was performed by a neurologist with expertise, as already described elsewhere [36]. The syringes used were disposable 1 mL ones and presented a 29 G needle; the quantity of drug to be administered was 5 IU (0.1 mL) on each site. We used the standardized follow-the-pain protocol, distributing the drug among 39 sites: procerus muscle (5 IU); 2 sites at the level of the corrugator muscles (10 IU); 4 sites across the frontalis muscles (20 IU); 10 sites across the temporalis muscles (50 IU); 8 sites across the occipitalis muscles (40 IU); 5 sites for the cervical paraspinalis muscles (20 IU); and, lastly, 50 IU injected across 10 sites of the horizontal trapezii muscles. Any immediate side effect was evaluated in the minutes after the therapy, while delayed ones were asked about during the following session.

### 4.5. Clinical Assessment

#### 4.5.1. Headache Features

Headache frequency and acute medication use data were collected through a specific headache diary provided to patients. Particularly, we investigated the number of MMDs (i.e., the days in which a patient complained of a headache with migraine characteristics or that led him/her to take an acute medication with its resolution) and the number of MADs (i.e., the days in which the patient took any acute medication for headache). Moreover, we distinguished patients as responders if they showed an MMDs reduction ≥ 30% from baseline and as nonresponders if the reduction from baseline was < 30%, following BoNT-A guidelines [42].

#### 4.5.2. Pittsburgh Sleep Quality Index (PSQI)

The PSQI [43] is a 19-item self-administered questionnaire that evaluates seven different components: sleep quality, sleep time, habitual sleep efficiency, sleep disorders, sleep medications, and daytime dysfunction in the last month. Scores range from 0 (excellent sleep quality) to 21 (severe impairment of sleep quality); a score >5 indicates that the patient reports poor sleep quality, while a score ≤5 is an index of good sleep quality [43]. The questionnaire has been widely used in migraine research to investigate the relationship between migraine and sleep quality [9,32].

#### 4.5.3. Psychological Symptoms Investigation

The authors decided to use two different questionnaires to explore the possible psychological symptoms in CM patients, namely, the HADS and the BDI-II.

##### HADS

HADS is a 14-item scale, specifically developed for outpatients, used as a screening tool to identify anxiety and depression symptoms [44]. The questionnaire provides two separate scoring systems for anxiety and depression; questions score from 0 (no symptom) to 3 (severe symptom); the total score ranges from 0 (normal) to 21 (severe). Scores up to 7 reflect normal values, while scores equal to or greater than 8 denote considerable symptoms of anxiety or depression. Such a questionnaire has been used to evaluate pain-related emotions in painful syndromes, including migraine [45].

##### BDI-II

BDI-II is a 21-item questionnaire that evaluates the impact of depression symptoms on daily life; questions score from 0 (no symptom) to 3 (severe symptom); the total score ranges from 0 to 63 and distinguishes 4 grades of depressive symptoms: 0–13 represents no depressive insight, 14–19 means mild symptoms, 20–29 means moderate ones, and 30–63 configures a severe depressive component. Compared to HADS, BDI-II can also distinguish between cognitive symptoms and somatic-affective ones [46].

#### 4.5.4. Headache Impact and Related Disability

Migraine represents the second cause of years lived with disability worldwide and the first one among people between the 2nd and 5th decades of life [47]; thus, several means to measure the impact and disability on patients’ lives have been designed. Authors chose to use the HIT-6 and MIDAS as reproducible questionnaires, universally accepted by the headache experts community, to assess and monitor the evolution of such a burdensome disease during follow-ups.

##### HIT-6

HIT-6 is a questionnaire containing 6 items, and it was created to investigate the impact that headache shows on patients’ everyday life. The test is demonstrated to be a convenient means to monitor migraineurs during follow-ups [48]. The questionnaire investigates the general perceived intensity of the headache and the detrimental effects on daily living. Moreover, some items investigate physical, work, or social aspects of life, in addition to the associated psychological distress. The six items are constituted by direct questions about the frequency of headache negative effects, and the patient is asked to give an answer which may be *never* (=6 points), *rarely* (=8 points), *sometimes* (=10 points), *very often* (=11 points), or *always* (=13 points); the total score ranges between 36 and 78, where higher scores indicate more impact on the patient’s life.

##### MIDAS

MIDAS is a simple questionnaire containing 5 items, and it is specifically designed for migraine patients; it represents a very useful means to assess the migraine-depending disability in a period of 3 months before the examination [49]. The test contains 5 questions that have the objective to quantify the number of days the patient lived with a certain disability due to migraine; in more detail, the items that are investigated are represented by paid work or schoolwork (days off and days when productivity is reduced by at least 50%, index of absenteeism and presenteeism, respectively), then household work (in the same manner); lastly, it considers even the leisure days or days of social activities lost because of migraine. The total score is obtained by summing the number of the days reported by the patient. It is then possible to distinguish 4 disability grades: I (0–5) *minimal* disability; II (6–10) *mild*; III (11–20) *moderate*; and IV (>20) *severe* disability in everyday life.

### 4.6. Statistical Analysis and Data Presentation

Qualitative data were reported as absolute frequencies (N) and percentages (%), while quantitative data were reported as mean ± SD. Statistical analyses were performed using IBM SPSS v.26 statistical software, and the changes between t0 and t1 were investigated through Kruskal–Wallis’s test for independent samples. Qualitative variables were evaluated using a chi-square test. Lastly, the correlation analysis was performed using the Spearman’s rank correlation coefficient including t0 and t1 data.

## Figures and Tables

**Table 1:** Demographic and general clinical features of the included patients.

**Variable**	**N**	**%**
Patients	42	100.0
Female	32	76.2
Male	10	23.8
Migraine without aura	40	95.2
Migraine with aura	2	4.8
**Variable**	**Mean**	**SD**
Age	49.3	10.2
Years from migraine onset	24.2	12.9
**Type of acute medication**	**N** ^1^	**%** ^1^
Triptans	27	64.3
NSAIDs	15	35.7
Association of indomethacin 25 mg, caffeine 75 mg, and prochlorperazine 2 mg	7	16.7
Betamethasone 4 mg ^2^	3	7.1
Acetaminophen	2	4.8

^1^ Note that the number of patients and the percentage exceed 42 and 100% since patients may have used the different types of medication in association, in temporal succession, or as alternatives; ^2^ vials for IM injection. Abbreviations: IM: intramuscular; N: number; NSAIDs: nonsteroidal anti-inflammatory drugs; SD: standard deviation.

**Table 2:** Headache frequency and acute drug use variation.

Variable	t0 Mean (SD)	t1 Mean (SD)	*p* Value
MMDs	20.6 (6.0)	13.6 (6.2)	<0.001
MADs	18.4 (7.7)	15.5 (10.1)	0.053

Abbreviations: MADs: monthly acute medication days; MMDs: monthly migraine days; SD: standard deviation.

**Table 3:** Sleep quality questionnaire scores.

Variable	t0 Mean (SD)	t1 Mean (SD)	*p* Value
PSQI	11.0 (5.0)	9.8 (4.6)	0.277

Abbreviations: PSQI: Pittsburgh Sleep Quality Index; SD: standard deviation.

**Table 4:** Psychological questionnaires scores.

Variable	t0 Mean (SD)	t1 Mean (SD)	*p* Value
BDI-II	16.7 (10.2)	15.7 (10.3)	0.678
HADS-a	10.3 (4.8)	9.3 (5.5)	0.492
HADS-d	7.2 (3.9)	7.1 (5.0)	0.901

Abbreviations: BDI-II: Beck Depression Inventory, 2nd edition; HADS: Hospital Anxiety and Depression Scale; HADS-a: anxiety subscale; HADS-d: depression subscale; SD: standard deviation.

**Table 5:** Impact questionnaires scores.

Variable	t0 Mean (SD)	t1 Mean (SD)	*p* Value
HIT-6	56.4 (12.5)	53.7 (17.5)	0.520
MIDAS	86.8 (64.4)	59.3 (42.9)	0.033

Abbreviations: HIT-6: Headache Impact Test 6; MIDAS: Migraine Disability Assessment; SD: standard deviation.

## Data Availability

Data will be available after reasonable request to the corresponding author.

## Supplementary Materials

The following supporting information can be downloaded at: https://www.mdpi.com/article/10.3390/toxins15090527/s1, Figure S1: Spearman correlation analysis of all the studied parameters.
