# Supplementary material for: Effects of a Single Session of OnabotulinumtoxinA Therapy on Sleep Quality and Psychological Measures: Preliminary Findings in a Population of Chronic Migraineurs
Source: Toxins (Basel). 2023 Aug 27;15(9):527. doi: 10.3390/toxins15090527 (PMC10537449; doi:10.3390/toxins15090527)
Supplement: Supplementary file 1 [file toxins-15-00527-s001.zip › toxins-2524952-supplementary.pdf]

Supplementary Materials: Effects of a single session of onabotulinumtoxinA therapy on sleep quality and psychological measures: preliminary findings in a population of chronic migraineurs

Angelo Torrente, Paolo Alonge, Laura Pilati, Andrea Gagliardo, Lavinia Vassallo, Vincenzo Di Stefano, Antonino Lupica, Irene Quartana, Giovanna Viticchi, Mauro Silvestrini, Marco Bartolini, Cecilia Camarda and Filippo Brighina

		MMDs	MADs	HIT-6	MIDAS	BDI-II	HADS-a	HADS-d	PSQI
MMDs	rho	1,000	,422**	,303**	,507**	,232	,114	,084	,443**
	Significance	.	,000	,007	,000	,218	,549	,658	,000
MADs	rho	,422**	1,000	-,183	,143	-,023	,029	-,093	,213
	Significance	,000	.	,112	,207	,908	,883	,636	,059
HIT-6	rho	,303**	-,183	1,000	,416**	,544**	,495**	,587**	,178
	Significance	,007	,112	.	,000	,002	,005	,001	,117
MIDAS	rho	,507**	,143	,416**	1,000	,518**	,486**	,588**	,540**
	Significance	,000	,207	,000	.	,003	,006	,001	,000
BDI-II	rho	,232	-,023	,544**	,518**	1,000	,866**	,804**	,772**
	Significance	,218	,908	,002	,003	.	,000	,000	,000
HADS-a	rho	,114	,029	,495**	,486**	,866**	1,000	,810**	,723**
	Significance	,549	,883	,005	,006	,000	.	,000	,000
HADS-d	rho	,084	-,093	,587**	,588**	,804**	,810**	1,000	,736**
	Significance	,658	,636	,001	,001	,000	,000	.	,000
PSQI	rho	,443**	,213	,178	,540**	,772**	,723**	,736**	1,000
	Significance	,000	,059	,117	,000	,000	,000	,000	.

Figure S1. Spearman correlation analysis of all the studied parameters. ** = correlation is significant if ≤ 0.01 (two tails). Abbreviations: BDI-II: Beck Depression Inventory, 2nd edition; HADS: Hospital Anxiety and Depression Scale; HADS-a: anxiety subscale; HADS-d: depression subscale; HIT-6: Headache Impact Test 6; MADs: monthly acute medication days; MIDAS: Migraine Disability Assessment; MMDs: monthly migraine days; PSQI: Pittsburgh Sleep Quality Index.
